# Bring it on: Top five antimicrobial stewardship challenges in transplant infectious diseases and practical strategies to address them

**DOI:** 10.1017/ash.2022.53

**Published:** 2022-04-27

**Authors:** Miranda So, Helen Tsai, Neeraja Swaminathan, Rachel Bartash

**Affiliations:** 1 Sinai Health-University Health Network Antimicrobial Stewardship Program, University Health Network, Toronto, Ontario, Canada; 2 Leslie Dan Faculty of Pharmacy, University of Toronto, Toronto, Ontario, Canada; 3 Division of Infectious Diseases, Department of Medicine, Montefiore Medical Center, Albert Einstein College of Medicine, Bronx, New York, United States

## Abstract

Antimicrobial therapies are essential tools for transplant recipients who are at high risk for infectious complications. However, judicious use of antimicrobials is critical to preventing the development of antimicrobial resistance. Treatment of multidrug-resistant organisms is challenging and potentially leads to therapies with higher toxicities, intravenous access, and intensive drug monitoring for interactions. Antimicrobial stewardship programs are crucial in the prevention of antimicrobial resistance, though balancing these strategies with the need for early and frequent antibiotic therapy in these immunocompromised patients can be challenging. In this review, we summarize 5 frequently encountered transplant infectious disease stewardship challenges, and we suggest strategies to improve practices for each clinical syndrome. These 5 challenging areas are: asymptomatic bacteriuria in kidney transplant recipients, febrile neutropenia in hematopoietic stem cell transplantation, antifungal prophylaxis in liver and lung transplantation, treatment of left-ventricular assist device infections, and *Clostridioides difficile* infection in solid-organ and hematopoietic stem-cell transplant recipients. Common themes contributing to these challenges include limited data specific to transplant patients, shortcomings in diagnostic testing, and uncertainties in pharmacotherapy.

Antimicrobial agents are life-saving medications for immunocompromised hosts who rely heavily on these medications. However, antimicrobial resistance (AMR) is a significant threat to these patients and, as such, judicious use of antimicrobials is critical. Antimicrobial stewardship (AMS) programs are essential in creating treatment guidelines as well as promoting and monitoring appropriate antimicrobial treatment in these complex patients. We discuss 5 important, frequently encountered transplant infectious disease stewardship challenges, with suggested strategies to address and improve antimicrobial practices for each clinical syndrome. Although each syndrome has its unique set of challenges, we discuss overarching themes of scarcity of data on stewardship interventions specific to transplant patients, limitations in diagnostic testing, and pharmacotherapy concerns.

## 1. Asymptomatic bacteriuria in renal transplant recipients

Asymptomatic bacteriuria (ASB), a common condition in renal transplant recipients, is often treated with antibiotics^1^ based on the theoretical risk of ascending infection leading to pyelonephritis and acute graft loss. However, data against routine treatment of ASB are fairly robust. The Infectious Diseases Society of America (IDSA) and the American Society for Transplantation (AST) updated their clinical care guidelines in 2019, recommending against treating ASB in renal transplant recipients >2 months after transplant.^2,3^ These guidelines reflected results from several limited retrospective studies^4–6^ and a single randomized controlled trial^7^ that found no significant differences in outcomes for patients who received antibiotics for ASB compared to those who did not. Following publication of these guidelines, additional randomized controlled trials and 1 meta-analysis have provided additional evidence that treating ASB does not offer benefit^8–10^ but leads to excessive antibiotic use and increased risk of infection with multidrug-resistant organisms.^8^ Despite strong evidence, ASB continues to be a stewardship challenge in renal transplant recipients.

Data on ASB outcomes in renal transplant recipients within 2 months of transplant or with anatomic genitourinary abnormalities, indwelling catheters, or ureteral stents, remains limited and guidelines do not make strong recommendations for these populations.^2,3^ Many providers may favor treating ASB in these circumstances^1^ because of concerns that foreign material, intense immunosuppression, or genitourinary tract abnormalities could potentiate the risk of ASB progressing to graft pyelonephritis, although the benefit is unclear.^10^

Stewardship efforts are further hindered by the diagnostic complexities of distinguishing ASB from urinary tract infections. In renal transplant recipients with nonspecific signs of infection but no clear urinary symptoms, positive urine cultures are difficult to interpret because denervation from surgery may limit the presence of urinary symptoms.^3,10^ Pyuria and positive urine cultures in the setting of chronic indwelling catheters or stents often reflect colonization or contamination rather than a UTI. Similarly, acute kidney injury and pyuria can be seen in both graft rejection and UTIs. Diagnostic ambiguities likely contribute to ASB overtreatment despite lack of clinical significance.

The use of other markers of infection (eg, white bold cell count, C- reactive protein level, and erythrocyte sedimentation rate) may be useful in differentiating asymptomatic bacteriuria from urinary tract infection in the setting of nonspecific clinical symptoms, but additional studies are needed. Improved biomarkers and diagnostic testing to discern the relevancy of positive urine cultures and to identify which renal transplant recipients would benefit from antibiotics can hopefully augment stewardship efforts in the future, but these are currently unavailable.

Moreover, whether ASB guidelines are reflected in clinical practice is unclear. In a survey study of European transplant centers, >70% reported routine screening for bacteriuria and treatment was common.^1^ One solution to limiting the treatment of ASB is avoiding routine surveillance cultures in the absence of symptoms or laboratory abnormalities because providers may be inclined to treat known positive results. Updating institutional treatment guidelines to include avoiding treatment and providing prescriber feedback are AMS tools that can help decrease the treatment of ASB.

## 2. Febrile neutropenia in stem-cell transplant recipients

Hematopoietic stem cell transplant (HSCT) recipients are vulnerable to infectious complications, especially during the pre-engraftment period in which significant neutropenia and mucosal damage increase the risk of bacteremia.^11–13^ HSCT recipients often receive several weeks of broad-spectrum antimicrobials to mitigate this risk. However, this extensive antimicrobial exposure, combined with prior antimicrobial therapy and conditioning chemotherapy, can contribute to gut dysbiosis and poor outcomes.^14,15^ AMR has been recognized as a leading cause of death globally, with carbapenem-resistant Enterobacterales (CRE) and vancomycin-resistant enterococci (VRE) emerging as major threats to HSCT recipients.^16–19^ Although AMS has been advocated for patients with hematological malignancies and HSCT, clinicians have to balance maintaining adequate antimicrobial coverage against minimizing unnecessary antimicrobial exposure at the individual and population level.^20–23^ Strategies to guide antimicrobial use in the HSCT population are urgently needed.

One area of stewardship interest is antimicrobial use for febrile neutropenia in HSCT recipients. Routinely, broad-spectrum coverage is maintained until absolute neutrophil count recovers to >500 cells/µL and the patient is afebrile, irrespective of the presence of a documented infection or fever of unknown origin.^24^ Antimicrobial prescribing practice in HSCT patients with febrile neutropenia varies widely; it informs and is informed by regional epidemiology.^25^

Recent data suggest that a shorter duration of antibiotic therapy is safe and effective for febrile neutropenia when coupled with close monitoring. The “How Long” study compared the conventional approach to high-risk febrile neutropenia with early discontinuation of broad-spectrum antimicrobials based on resolution of fever after 72 hours and clinical recovery.^26^ Patients in the experimental arm received a significantly shorter duration of antimicrobials with numerically fewer adverse events, indicating that the symptom-driven approach avoided unnecessary antimicrobial exposure.^26^ Although this study included HSCT patients, allogeneic recipients accounted for only 9% of the study population.^26^ Nevertheless, these findings show that in fever of unknown origin, shortening duration of empirical antimicrobial therapy with close monitoring can be safe and feasible. Two recent studies with a similar intervention and a larger representation of HSCT patients reported congruous findings, but well-designed, prospective studies are imperative to supporting practice changes in the era of AMR.^27–29^

In patients with documented infections, the decision to tailor therapy targeting the pathogen versus continuing with broad-spectrum antimicrobials has not been fully elucidated. One guideline recommended a patient-specific approach, which may lead to wide variability in antimicrobial prescribing.^30^ A multinational, prospective, longitudinal study of patients with high-risk febrile neutropenia, including acute leukemia and HSCT (autologous and allogeneic) recipients, evaluated the association between bacteremia and mortality at 7 days and 30 days.^31^ *P. aeruginosa* bacteremia was associated with the highest 7-day and 30-day mortality at 16.7% and 26.7%, respectively, compared to coagulase-negative staphylococci (2%) or streptococci (<1%), whereas enterococci were associated with an unexpected increase in mortality.^32^ Candidemia and gram-negative bacteremia were independently associated with intensive care unit admission.^32^ In a related study, predictors of bacteremia due to multidrug-resistant *P. aeruginosa* included prior exposure to piperacillin-tazobactam, antipseudomonal carbapenem, fluoroquinolone prophylaxis, underlying hematological disease, and presence of a urinary catheter.^33^ These data suggest that a pathogen-specific approach combined with judicious use of broad-spectrum antimicrobials may be optimal. Although prophylactic fluoroquinolones have been routinely recommended for patients with acute leukemia and HSCT to reduce the rates of bacteremia, a more thoughtful, risk-stratified approach should be considered given its implications for institutional epidemiology.^15,34–36^

Implementing an institution-specific guideline for management of neutropenic fever in hematology-oncology patients that accounts for local susceptibility patterns is a recommended AMS intervention.^37,38^ To ensure sustained practice change, evaluating the quality of antimicrobial prescribing through auditing and provision of feedback to prescribers will help AMS programs identify areas for improvement and provide ongoing support for guideline-adherent practices in hematology patients and HSCT recipients.^39^ In the management of high-risk febrile neutropenia, data supporting judicious prescribing with close monitoring of patients with fever of unknown origin are encouraging. A multipronged approach with risk stratification, implementation of local guidelines, and evaluation of quality of antimicrobial prescribing offers a potential solution that may overcome the challenges of AMS in this vulnerable patient population.

## 3. Antifungal prophylaxis in liver and lung transplant recipients

Antifungal prophylaxis has been advocated for SOT recipients because diagnostic limitations for invasive fungal infections can translate into treatment delays that confer significant morbidity and mortality.^40^ Stewardship challenges involving antifungal prophylaxis in liver transplant recipients include pharmacokinetic considerations and the availability of local epidemiological patterns of fungal infections. In lung transplant recipients, inhaled amphotericin or systemic azole therapy are used because invasive aspergillosis is a significant concern. However, stewardship efforts are hindered by the absence of strong evidence, and a better understanding of risk is needed.

Targeted rather than universal antifungal prophylaxis based on a risk stratification approach outlined by the AST is preferred for liver transplant recipients, but prospective studies to guide treatment duration are lacking.^41^ Withholding antifungal prophylaxis in low-risk liver transplant recipients has been shown to be safe, to reduce unnecessary exposure, and to avoid potential drug–drug interactions with immunosuppressants.^41,42^ In a 2008 study, 28% and 72% of North American transplant centers surveyed used universal and targeted prophylaxis, respectively.^43^ More recent data on prescribing trends are lacking, but regular institutional review of prescribing patterns in low-risk recipients is a realistic AMS tool to ensure that evidence-based practice is followed and that antifungal overuse is limited.

The various antifungal agents available for targeted prophylaxis in high-risk liver transplant recipients have notable limitations. Liposomal amphotericin B is effective,^44^ but it offers unnecessarily broad coverage, it is costly, and it is limited to intravenous administration. Fluconazole, the preferred agent based on expert opinion,^41^ is faced with rising resistance, increasing rates of non-*albicans Candida* spp infections,^45^ and known interactions with calcineurin inhibitors. A single-center study demonstrated that fixed fluconazole dosing was effective, and no invasive fungal infections with reduced fluconazole-susceptible strains occurred.^46^ However, applicability across institutions with different local epidemiologies and among critically ill patients with renal dysfunction (in whom fluconazole pharmacokinetics are variable) are concerns.^47^ Echinocandins are associated with fewer toxicities and drug–drug interactions, but there are significant pharmacokinetic–pharmacodynamic limitations. Echinocandins achieve limited therapeutic concentrations intra-abdominally because of their molecular characteristics,^48^ predisposing patients to the emergence of echinocandin resistance.^49^ An 8% acquired resistance rate^50^ and breakthrough invasive fungal infections^48^ while on echinocandin therapy have been noted. To help prevent the emergence of fluconazole and echinocandin resistance, AMS programs can assist in optimizing dosing for patients who are critically ill, who require renal replacement therapy, or who have infections at sites of known poor drug penetration.^47,48^ The risk of invasive aspergillosis in high-risk liver recipients is another crucial consideration.^51^ The decision to administer antimold coverage involves weighing the local incidence of and a recipient’s risk for invasive aspergillosis^52^ against potential toxicities, drug–drug interactions, and emergence of azole-resistant *Aspergillus*. This challenging situation underscores the importance of updated local epidemiological data and of longitudinal monitoring of fungal susceptibilities, outcomes, and adverse events to form AMS strategies that can be feasibly adopted in clinical practice.^53,54^

For lung transplant recipients, the preferred choice between universal or pre-emptive antifungal prophylaxis against *Aspergillus* (the latter involving routine surveillance with broncho-alveolar lavage culture and galactomannan) is undefined, but either approach is recommended over no prophylaxis.^55,56^ Although 90% of US transplant centers had previously reported routine universal antifungal prophylaxis for lung transplant recipients, a review of administrative claims data showed that only 41.5% of patients received antifungal prophylaxis.^57^ The reasons for this incongruence is unknown, but further analysis could potentially provide insights to stewardship areas of interest. An understanding of the current practices would help direct AMS efforts toward high-yield measures. Data on universal and pre-emptive therapies are mixed because studies are limited by small sample size, variable study design, and heterogeneous immunosuppression and antifungal agents included.^58^ Prospective studies comparing universal and pre-emptive prophylaxis are needed not only to evaluate efficacy but also to characterize potential stewardship benefits of pre-emptive prophylaxis.

If universal prophylactic therapy for lung transplant recipients is employed, the recommended duration of prophylaxis is 4–6 months. Nevertheless, 22.2% of transplant centers in one survey continued universal prophylaxis for >12 months.^59^ The use of long-term or lifelong azole prophylaxis has not been shown to alter the incidence of invasive fungal infection in lung transplant recipients, even in the setting of therapeutic azole levels, and it is associated with medication toxicities, healthcare costs, and potential resistance.^60^ Another area of stewardship concern is whether routine prophylaxis is driving the emergence of delayed aspergillosis and invasive fungal infections in lung transplant recipients after prophylaxis is discontinued, beyond the traditional risk period.^61,62^ One center reported the median time of onset for invasive aspergillosis in lung transplant recipients to be 363 days.^61^ The incidence and consequences of invasive aspergillosis occurring beyond the first year have not been clearly established, even though immunosuppression may be less intensive and the risk of anastomotic fungal infection or ulcerative tracheobronchitis may be lower.

Perhaps a more tailored approach to antifungal prophylaxis in lung transplant recipients is necessary. Evidence-based risk stratification models to identify recipients who would benefit from a short course versus a standard course or from lifelong antifungal prophylaxis, relative to local incidence of invasive fungal infection, would be valuable for AMS programs. We also suggest that AMS programs monitor closely for a potential risk in delayed invasive aspergillosis and that they analyze any occurrences because such cases will have significant local and population-level stewardship implications.

## 4. Left ventricular assist device infections

Infections, a leading complication of left ventricular assist devices (LVADs), are estimated to occur in nearly 40% of recipients.^63^ Despite the high incidence, management guidelines for LVAD infections are based on observational data and expert opinion due to the absence of randomized controlled trials.^64^ The lack of strong evidence, along with diagnostic complexities and uncertain effects of LVADs on antimicrobial pharmacokinetics, contribute to the challenges facing AMS in LVAD-specific and related infections. Compounding these issues are the nature of the infections, which are potentially incurable without source control through transplantation, and the growing proportion of LVADs implanted for destination therapy.^65^ In 2019, 73.1% of LVADs implanted were for destination therapy.^65^ Because LVADS are increasingly used for destination therapy, these infectious complications will be a growing challenge for AMS.

Device driveline infections, which account for 12%–35% of all LVAD-specific or related infections^63^, occur most frequently but diagnosing and distinguishing superficial from deep infection is problematic. Clinical and physical exam features of driveline or endovascular infections can be subtle, nonspecific, or absent.^66,67^ Imaging to assist in diagnosis is not standardized and has limitations: Computed tomography has variable performance and is affected by device artifact, ultrasound detects only superficial fluid collections, and access to FDG-PET may be a barrier,^67^ although gallium single-photon emission computed tomography (SPECT) appears to be a promising imaging modality.^68^

Even if the extent of infection is successfully diagnosed, uncertainties remain regarding duration of therapy and the role of chronic antimicrobial suppression (CAS). The evidence for the current treatment duration recommendations for superficial and deep driveline infections is limited^64,67^ and, in clinical practice, widely variable.^67^ Whether superficial driveline infections progress to deeper infections or if CAS for driveline infections significantly reduces recurrence remains to be determined. Conflicting data are likely driven by the various LVAD-specific infections included in each study. Several studies estimate a 30% failure rate of CAS,^69–71^ with recurrence even in superficial driveline infections.^70^ One study has suggested that CAS resulted in no significant difference in the proportion of patients with relapse.^71^ For an infection associated with foreign material that may not be reasonably removed and that can occur in LVAD recipients who have altered immune responses,^67^ the implications of these retrospective data on clinical practice are unclear.

In addition to ongoing questions regarding duration of therapy for LVAD infections, 2 studies have suggested altered intravenous vancomycin pharmacokinetics from LVADs, which may further complicate AMS efforts in these patients. Those with LVADs had a significantly higher incidence of supra-therapeutic trough levels, potentially due to an overestimated volume of distribution and rate of elimination.^72,73^ Further characterization of this finding and its implications are important because *S. aureus* and coagulase-negative *Staphylococcus* are the predominant etiological agents of LVAD infections.

The challenges facing AMS in LVAD infections are driven by the need for improved diagnostics and well-designed studies on treatment. In the absence of heart transplantation, determining the therapy end point is complex and requires careful consideration of a patient’s clinical, microbiological, radiographic, and surgical factors. The role for CAS remains ambiguous due to the limited evidence on efficacy and adverse effects. As more individuals receive LVADs for destination therapy, studies describing long-term outcomes of CAS categorized by each type of LVAD infection and pathogen involved are needed to assist in optimizing antimicrobial use.

## 5. *Clostridioides difficile* infection

*Clostridioides difficile* infection (CDI) is a major cause of morbidity and mortality that disproportionally affects HSCT and SOT recipients. Compared to the general inpatient population, HSCT and SOT patients have a higher incidence of CDI, are more likely to have severe infection, and are at greater risk of recurrence.^74^ For these reasons, reducing CDI rates is a priority of AMS programs. Interventions aimed at restricting antimicrobial exposure and providing provider education and feedback have been highly successful.^75,76^ Effective CDI antimicrobial stewardship practices are an interdisciplinary effort engaging diagnostic stewardship and infection prevention and control.^74,76^ Active adaptation of these practices to the dynamic and unique factors of each transplant center is crucial for sustained progress in reducing CDI burden in this population. Stewardship areas of uncertainty include appropriate patient selection for testing, implications of asymptomatic screening, and the role of anti-CDI therapeutic prophylaxis.

The use of multistep testing algorithms has improved the analytic diagnostic stage, but CDI is a clinical diagnosis that depends on preanalytic decisions.^76^ Differentiating asymptomatic colonization from CDI is a long-standing diagnostic conundrum that is particularly problematic in the transplant population. Transplant patients are at risk of overdiagnosis because of increased risk of toxigenic *C. difficile* carriage^77^ and multiple confounding factors that cause diarrhea, including antibiotic use, immunosuppressive medications, mucositis, and graft-versus-host disease.^78^ Comprehensive review for other etiologies of diarrhea relative to a patient’s clinical symptoms such as degree of diarrhea, should be a priority prior to testing.^79^

Screening transplant patients at admission for *C. difficile* colonization facilitates early implementation of infection prevention measures and may result in decreased horizontal transmission.^78,80^ In one study of patients admitted to an inpatient hematological unit, colonization with *C. difficile* conferred an 11.6 times higher odds of progression to CDI compared to those without colonization.^81^ Identification of these asymptomatic carriers is an opportunity for targeted risk-reduction measures, such as antimicrobial review, to reduce the risk not only for symptomatic infection but for vertical transmission as well. However, the role of prophylactic, pharmacological measures to prevent the progression of colonization to infection is unknown. It is unclear how to optimally use asymptomatic screening for AMS efforts without unintentionally causing inappropriate treatment from misinterpretation of tests.^80,82^

Oral vancomycin prophylaxis is an attractive option to prevent CDI. Retrospective reviews suggest that primary prophylaxis in allogenic HSCT recipients is associated with significantly lower rates of CDI^83,84^ and that secondary prophylaxis is effective in reducing CDI recurrence in kidney transplant patients.^85^ Although these studies found no instances of vancomycin-resistant *Enterococcus* (VRE) colonization or bacteremia,^83,84^ several other studies have found that the use of oral vancomycin increased the risk of VRE overgrowth and infection^86,87^ and can alter gut microbiome, which is linked to poor outcomes.^79^ Despite the suggested benefit, a number of questions on the potential role for prophylaxis remain, including optimal duration, cost implications, effects on the intestinal microbiome, potential to drive the emergence of vancomycin-resistant *C. difficile* strains, and the benefit of secondary prophylaxis if fidaxomicin was used as initial therapy. Additional research can help characterize factors that may portend increased risk of CDI in HSCT and SOT recipients to build a risk stratification approach to prophylaxis.

In conclusion, the challenges facing AMS in transplant infectious diseases illustrate the difficulties in integrating the available evidence and diagnostic uncertainties with host-specific and local epidemiological factors to implement measures catered to both individuals and larger populations. The challenges, which are summarized in Table 1, have shared features, but specific solutions vary and should be personalized to institutional epidemiological patterns. In these uniquely vulnerable hosts, there is no “one size fits all” approach to AMS. For AMS challenges supported by strong evidence, we suggest that the implementation of practices in context of local epidemiology and dynamic evaluation over time to develop sustained, targeted measures. For challenges driven by knowledge gaps, recognizing the limitations of current evidence and engaging interdisciplinary teams to help risk-stratify patients are important to inform clinical practice. Employing thoughtful strategies is crucial for this population, which is disproportionally affected by infections and at risk for adverse effects of antimicrobial misuse and overuse.

**Table 1.** Summary of the Top 5 Antimicrobial Stewardship Challenges and Potential Mitigating Strategies

Syndrome	The Challenge	Mitigating Strategies
Asymptomatic bacteriuria (ASB) in kidney transplant recipients	– Scarce data to guide approach to ASB in renal recipients within 2 months posttransplant and those with anatomic GU abnormalities, indwelling catheters, or ureteral stents – Diagnostic challenges in discerning ASB from infection or rejection contribute to unnecessary antimicrobial prescribing	– Use biomarkers such as C reactive protien and erythrocyte sedimentation rate to differentiate ASB from infection. – Avoid routine surveillance urine cultures in the absence of symptoms or abnormal labs. – Offer prescriber feedback.
Febrile neutropenia in hematopoietic stem cell transplant (HSCT) recipients	– Broad-spectrum antimicrobials are often maintained until absolute neutrophil count >500 cells/μL, irrespective of presence of documented infections or fever of unknown origin because patients are at high risk of sepsis. – Prolonged exposure to antimicrobial therapy contribute to gut dysbiosis, poor outcomes, and multidrug-resistant pathogens	– Emerging data suggest safety of stopping broad-spectrum antimicrobials with close monitoring in stable patients even if absolute neutrophil count is <500 cells/μL. – Follow a pathogen-specific approach to risk stratify duration of antimicrobial therapy in bacteremia. – Implement institution-specific guideline for febrile neutropenia in HSCT and hematology-oncology patients. – Evaluate quality of antimicrobial prescribing and adherence to guideline.
Antifungal (AF) prophylaxis	– No antifungal prophylaxis is appropriate for low-risk liver transplant recipients. – Rising prevalence of fluconazole resistant *C. albicans* and nonalbicans *Candida* spp and echinocandin resistance – Echinocandins achieve low therapeutic concentrations in critically ill patients and infection sites of interest – Data comparing universal and pre-emptive prophylactic strategies in lung transplant recipients are limited. – Antifungal prophylaxis prescribing patterns for lung transplant recipients vary.	– Evaluate adherence to guidelines and offer provider feedback. – Utilize local antifungal infection and susceptibility patterns and engage providers to optimize fluconazole and echinocandin dosing. – Design evidence-based risk stratification models to identify optimal antifungal course for recipients.
Left ventricular assistive device (LVAD) infections	– LVADs are increasingly used for non–heart-transplant candidates as destination therapy. – Diagnostic difficulties in distinguishing superficial versus deep drive-line infections – LVADs alter vancomycin pharmacokinetics. – Data to guide duration of antimicrobial therapy and the role of chronic antimicrobial suppression, are limited.	– Implement therapeutic drug monitoring for vancomycin may minimize toxicity. – Perform comprehensive assessment of clinical, diagnostic, and surgical factors to determine duration of therapy. – More data are required to guide antimicrobial decisions.
*C. difficile* infections (CDI)	– CDI causes significant morbidity and mortality in HSCT and SOT recipients. – Diagnostic challenges in differentiating infection from colonization in the context of medications, mucositis, immunosuppressants and graft-versus-host-disease remain a concern. – Optimal testing strategy, implications of asymptomatic screening and the role of prophylaxis are key uncertainties.	– Engage multidisciplinary teams to implement coordinated interventions to manage CDI. – Balance potential benefits of primary or secondary prophylaxis with the risk of emerging antibiotic-resistant pathogens.

Note. HSCT, hematopoietic stem cell transplant.
